# A Qualitative Research Study Which Explores Humanitarian Stakeholders’ Views on Healthcare Access for Refugees in Greece

**DOI:** 10.3390/ijerph17196972

**Published:** 2020-09-23

**Authors:** Liz Joseph, Sharif A. Ismail, Meghan Gunst, Kate Jarman, Dina Prior, Matthew Harris, Aula Abbara

**Affiliations:** 1 Department of Medicine, Imperial College, London SW7 2AZ, UK; liz.joseph14@imperial.ac.uk (L.J.); sharif.ismail15@imperial.ac.uk (S.A.I.); m.harris@imperial.ac.uk (M.H.); 2 Faculty of Public Health, London School of Hygiene and Tropical Medicine, London WC1H 9SH, UK; 3 Department of Paediatrics, Sydney Children’s Hospitals Network, Sydney, NSW 2031, Australia; meghan@gunst.com.au; 4 Independent Consultant in the International Health Sector, London, UK; kate.e.n.jarman@gmail.com (K.J.); dina.prior@me.com (D.P.)

**Keywords:** Greece, refugees, Syria, healthcare access, humanitarian, coordination

## Abstract

*Introduction:* As of January 2020, 115,600 refugees remain in Greece; most are Afghani, Iraqi or Syrian nationals. This qualitative research study explores the views of key stakeholders providing healthcare for refugees in Greece between 2015 and 2018. The focus was on identifying key barriers and facilitators to healthcare access for refugees in Greece. *Methods:* 16 interviewees from humanitarian and international organisations operating in Greece were identified through purposive and snowball sampling. Semi-structured interviews were conducted between March and April 2018. Data were analysed using the Framework Method. *Results:* Key themes affecting healthcare access included the influence of socio-cultural factors (healthcare expectations, language, gender) and the ability of the Greek health system to respond to existing and evolving demands; these included Greece’s ongoing economic crisis, human resource shortages, weak primary healthcare system, legal barriers and logistics. The evolution of the humanitarian response from emergency to sustained changes to EU funding, coordination and comprehensiveness of services affected healthcare access for refugees. *Conclusion:* The most noted barriers cited by humanitarian stakeholders to healthcare access for refugees in Greece were socio-cultural and language differences between refugees and healthcare providers and poor coordination among stakeholders. Policies and interventions which address these could improve healthcare access for refugees in Greece with coordination led by the EU.

## 1. Introduction

Since 2015, protracted conflicts have driven some of the largest flows of forced displacement since World War II, leading to more than one million refugees entering Europe mostly through Greece. As of January 2020, there are 115,600 refugees and migrants in Greece (41,200 of whom are on the Greek islands) [1] with the most common nationalities being Syrian, Afghan and Iraqi. Women account for 36.5% and children for 23% of the refugee population respectively [1]. Now in its ninth year, the Syrian conflict has been one of the most important drivers of refugee arrivals to Greece. More than half of the country’s 22 million strong pre-war population has been displaced, mostly as internally displaced persons. Externally, 5.6 million Syrians are now refugees hosted mainly in neighbouring countries including Lebanon, Jordan and Turkey [2]. The protracted nature of the conflict, poor labour market opportunities and increasingly uncertain political climate in these countries has driven many Syrians to seek refuge in Europe [3]. Though the journey from the western coast of Turkey to the Greek islands is short, it can be dangerous with smugglers using unfit-for-purpose, overcrowded vessels and sometimes fake life jackets to maximise profits [4]. Although we know that barriers to healthcare access for mobile populations can be profound, healthcare access for refugees and migrants in Greece is sparsely represented in literature. Barriers to healthcare access for refugees include those related to language, geography (distance of camps to healthcare facilities) and challenges navigating health systems which may differ from their own as well as language barriers. This study aims to help address this deficit. For the purposes of this manuscript, we use the internationally agreed UNHCR (United Nations High Commissioner for Refugees) definition of the term refugee [5].

### 1.1. Refugees in Greece Since the EU–Turkey Deal

Refugee flows into Greece reduced dramatically after the EU–Turkey deal in March 2016. This agreement promised [6] funds to Turkey and relocation of registered refugees from Turkey in return for accepting the return of refugees from Europe [7]. This agreement was criticised for what was perceived as a trade in refugees. As a result of the agreement, daily arrivals dropped from 10,000 per day in 2015 to less than 74 per day in March 2016 and continued to diminish into 2017. As of 2019, refugee arrivals have increased, though not to the levels seen in 2015 [8]**.** For Syrian and Afghani refugees, this increase likely reflects more challenging conditions in Turkey including deportations to unsafe conditions in Syria as well as increasingly challenging conditions in Iran, where some had previously sought refuge [9].

In March 2016, the intended route of refugees through Greece to Northern Europe became obstructed by the closure of the Greece-FYROM (Former Yugoslav Republic of Macedonia) border. As a result, large informal camps formed near, the largest of which were at Idomeni and Eko; these were both forcibly closed in 2016 [10,11]. Following closures, refugees were relocated to over 50 camps across mainland Greece and its islands, in which living conditions have become increasingly difficult. In May 2016, there were 58,130 refugees in Greece [1]. As of January 2020, this has increased to 115,600 [12] refugees including 13,800 in Moria camp (on Lesbos Island) where conditions fail to meet basic humanitarian standards, and refugees occupy a space intended for 3000 [13].

Harsh living conditions are contributing to a shift in health needs among refugees in Greece from predominantly communicable diseases, injuries and mental health needs early on [14] to more non-communicable diseases and chronic mental health complaints the longer they remained in camps in Greece. The uncertainty which refugees faced in Greece together with prior psychological traumas (e.g., incarceration, torture, discrimination) and the dire living conditions have resulted in significant psychological morbidities which have been challenging to be managed in Greece [15,16,17,18]. Meeting the needs of refugees both in Greece and elsewhere in Europe has proven challenging with uneven availability of services, impacts of migration regulation and restrictions on movement e.g., from islands to mainland Greece, difficulties navigating the health system and insufficient interpreters and cultural mediators [19]. Additional challenges include the heterogeneous mix of refugees from different countries with differing health priorities and different experiences or expectations of healthcare [20].

### 1.2. Rhetoric and Reality Concerning Access to Healthcare for Refugees in Greece

The availability and accessibility of health services to help meet the needs among refugee populations identified have changed over time, but barriers to healthcare access remain. Refugees in Greece could (until July 2019) obtain an AMKA number (Αριθμός Μητρώου Κοινωνικής Ασφάλισης - healthcare and social insurance number) and, theoretically, access free healthcare through the public health system. However, the practical level of healthcare access this actually gave refugees is questionable given the adverse effects of the ongoing economic crisis which has affected Greece’s health system, leaving it ill-resourced to meet the needs of its own population [18,20,21]. In July 2019, distribution of AMKA stopped. While the Greek government announced, in November 2019, a new law which would allow a Temporary Healthcare and Social Insurance Number for Alien Citizens for asylum seekers, this has not been put into action [22]. 

Academic literature which explores healthcare access for refugees in Greece is sparse [18,20,21,22,23,24,25], but existing work suggests that refugees continue to face challenges accessing healthcare which meets their needs. The aim of this qualitative research study was to examine the reality of healthcare access for refugees in Greece, and how this has evolved over time, from the perspective of those who have worked with humanitarian organisations and international organisations (e.g., UNHCR) between 2015 and 2018. We set out to understand the following:

1. What are the key barriers or facilitators for healthcare access for refugees in Greece (from the perspective of healthcare providers);
2. What challenges are faced by providers of healthcare for refugees in Greece when delivering their services;
3. The evolution of the health response for refugees in Greece between 2015 and March 2018.

## 2. Materials and Methods

A literature review to identify key themes was followed by a qualitative study in which key informants were interviewed and transcripts were thematically analysed using the Framework Approach [26]. This section sets out procedures for participant sampling and recruitment, conduct of the interviews, and finally their analysis. More details of the method are available in Appendix A.

### 2.1. Sampling and Participant Recruitment

Purposive sampling was used to identify key informants (KIs) who worked on the healthcare response in Greece including local and international NGOs and international organisations (e.g., UNHCR, WHO, UNICEF) in Greece, drawing on the authors’ personal contacts. Snowballing was then used to identify further potential KIs. KIs were selected for their involvement in the health response for the refugee crisis in Greece at any time since 2015. KIs were contacted by phone or email to recruit them to the study; one further contact attempt was made if there was no response. All interviews were conducted between March and April 2018 by the lead author. In person interviews were performed in person in Athens and in the UK, otherwise interviews were performed via Skype or phone. Verbal consent was recorded at the beginning of each interview and included permission for the recording and analysis of the interview content. Interviews lasted 1–2 h.

### 2.2. Key Informant Interviews

Interviews were conducted in accordance with a semi-structured interview topic guide (see Appendix B). Information about the KI’s place of work and experience was obtained. The interviews explored the following aspects of healthcare access for refugees: [1] changes to the healthcare response for refugees in Greece between 2015 and March 2018, [2] barriers or facilitators to accessing healthcare, [3] role of NGOs and international agencies in the health response and [4] gaps in healthcare provision for refugees in Greece.

### 2.3. Data Management and Storage

KIs were anonymised and numbered to protect confidentiality. All interviews were recorded and stored on a password protected device. The interviews were transcribed into MS Word and stored in password protected files.

### 2.4. Thematic Analysis

The interviews were analysed thematically using both deductive and inductive coding in Microsoft Word. Initial themes elicited from the literature and the Framework Method were adopted to perform the thematic analysis. The interviewer [L.J.] familiarised themselves with each of the transcripts as per the Framework Method [26] and then systematically reviewed the transcripts for recurring codes to develop further themes not originally identified in the literature. Codes were identified under each theme to highlight important aspects. Codes were examined, and a final analytical framework was developed once all interview transcripts had been coded. The framework was applied to the transcripts and relevant parts of the text were highlighted. Each of the themes and codes were then analysed until saturation had been reached.

### 2.5. Ethics

Formal ethics review was not required for this work as the interviewees were not refugees but were professionals. Even so, measures to maintain confidentiality including anonymity, secure data storage and consent were implemented rigorously. 

## 3. Results

A total of 16 KIs participated in the study, as characterised in Table 1.

**Figure 1:**
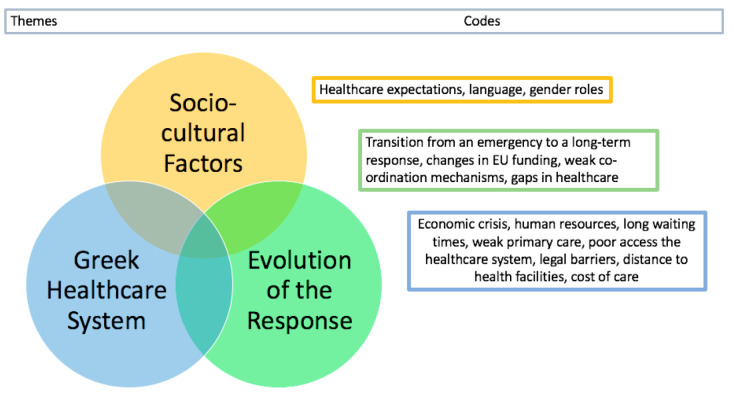
Main themes and codes emerging from analysis of key informants (KIs).

The main emerging themes are summarised in Figure 1. The following sections describe these themes and underlying codes in more depth.

### 3.1. Socio-Cultural Factors 

#### 3.1.1. Gender and Cultural Sensitivity of Care Provision

Most refugees were Muslim or Yazidi, with religious traditions and customs that were unfamiliar to many Greek healthcare workers. During Ramadan, Greek doctors might “face the issue of people not wanting to take their medications 3 times a day”, which they had not encountered before. One KI reflected:

*Many of the doctors and other staff are not used to working [with] such a multi-cultural, multi-national patient load so they have not been trained on how to do culturally sensitive medicine. This is another barrier for both of them to communicate effectively.*—KI 9

Two KIs reported that refugees may face stigma in Greece related to religion; however, in contrast, another noted that barriers related to cultural factors were not “*as extensive as you may think”*.

Some KIs noted that gender roles affected healthcare access among some refugee groups in a way that differed from the host community. One KI noted:

*Very frequently there are women who are reluctant to be examined by a male physician or there are men who are reluctant to be examined by a female. Also, for some women, the fact that the decision of whether they access healthcare is made by the male member of the family may restrict their access.*—KI 9

#### 3.1.2. Language

A number of KIs noted language and the lack of translators to be a critical barrier to healthcare access for refugees. Most refugees spoke Arabic, Kurdish (Kurmanji) or Pashtun, but translators were infrequently available. English was the most commonly spoken language among the health professionals working with refugees; as such, there was heavy reliance on translators, with consultations dropping when they or Arabic speaking staff were not present. This was mentioned frequently as a key issue which affected the ability of doctors to perform Primary Healthcare (PHC) consultations and onward referrals as well as affecting the quality of care received by the refugees. KIs noted that some refugees felt their concerns were dismissed as a result of language barriers and were unable to navigate transport systems to reach secondary care appointments. 

The impact of this was summed up by one KI:

*With translators our team can see around one hundred patients per day. But without, it can be as low as 20 per day.*—KI 1

A further concern was the number of languages spoken by the refugee population. KI 9 discussed the difficulty of finding enough people fluent in both English and Arabic (or English as well as another popular language amongst refugees) and its negative impact on quality of care. This challenge was highlighted by KI below:

*If sent by themselves with no translator, they [refugee patients] don’t know what’s going on and may find it difficult to transfer medical records back. Therefore, you lose a lot of valuable information. They also don’t know how to interact with the system effectively and advocate for their own needs.*—KI 2

#### 3.1.3. Healthcare Expectations

Participants reported that refugees, particularly those from Syria, were accustomed to high quality and accessible healthcare before the war, that featured secondary care, and consequently had high expectations of the healthcare provided in Greece. This led to frustration among Syrian refugees when their consultation was with a generalist rather than a specialist. One KI spoke of Syrian refugees’ expectations:

*They did not like seeing a primary health care physician; they would rather see a specialist as this was the system they were used to before the war. If you had chest pain, you would see a cardiologist. There was no GP system.*—KI 1

Some refugees were also accustomed to lower prescription thresholds or less regulated medication dispensing systems, in particular for antibiotics; this led to frustration as antimicrobial stewardship was stricter in Greece than in their countries of origin.

*Some of the refugees are very used to increased antibiotic prescriptions and although in Greece more antibiotics are prescribed than other European countries, they are still not satisfied if they do not get priority. For some of them, they are used to getting medications such as injections—in Greece injections are not very popular with doctors. So some are not satisfied for reasons of different medical cultures.*—KI 9

### 3.2. The Greek Health System

#### 3.2.1. Greece’s Economic Crisis

KIs noted that the protracted economic crisis in Greece complicated provision of healthcare for host communities, with some suggesting that the “collapsing” system required “support and restructuring”. In some ways, this makes the humanitarian context within Greece unique, as KI 15 reflected, *“one particularity of this context is that it coincides with a prolonged economic crisis”*. This affected the perception of some local communities who noted that free healthcare was offered to refugees while they had to make out of pocket contributions. Existing gaps for Greek nationals in the healthcare system were described:

*Not everyone has access, you are not always able to find the speciality you want, it can take months to get an appointment, there is a big waiting list.*—KI 5

#### 3.2.2. Human Resources for Health

Economic distress has contributed to “brain drain” in Greece as many healthcare workers, particularly nurses, have emigrated to seek stable and profitable employment. Some KI’s commented that the remaining system appeared chronically understaffed with low morale. KI 12 noted that this exodus was not unusual in countries experiencing economic crises. Samos, an island with a population of 2000 only had 1 doctor before the crisis and recruitment to this and similar areas was difficult.

*It was hard to find people that were willing to go to particular areas. Obviously, people want to live in big cities, but it is hard to get people to relocate to an island.*—KI 14

In addition to inconsistently and insufficiently staffed local services, volunteer healthcare workers and support staff were burdened and prone to burnout. KI 7 felt that their contribution was small and could not change the legal or residential status of the refugees. This situation was described as *“heart-breaking”* by KI 14 and affected their perceptions of care provision.

#### 3.2.3. Insufficient Access to Primary Care

In both Greece and the country of origin of many refugees, the primary healthcare (PHC) system was underdeveloped with heavy reliance on secondary care. KIs, particularly those working for NGOs who provided much of the PHC on the islands and camps noted that the Greek HS was *“very hospital orientated”*. KI 13 stated that general practitioner (GP) roles in Greece were unattractive due to their lower prestige and salaries compared to other specialties, making recruitment challenging. This may be related to the recent introduction of formal GP training in Greece. Additionally, the secondary care sector in Greece had been unregulated in terms of supply, community coverage and fees charged in the lead up to the refugee crisis [27]. One KI noted that improvements in PHC, which should include refugees in a universal healthcare scheme, could yield improvements:

*If you can do good primary healthcare, everything else becomes easier, you do not need really strong referral services to secondary care if you do good primary health care.—*KI 15

#### 3.2.4. Long Waiting Times for Secondary Care Appointments

Long waiting times to see health professionals in the Greek HS were noted, and this was also the case for refugees. KIs highlighted this as a cause of frustration for refugees who may not be used to such delays in their own health systems. This was noted to impact patient satisfaction, especially when a specialist referral could mean a waiting time of 2-3 months. Some refugees may *“wait all day and then only get five minutes of someone’s time”.*—KI 11

#### 3.2.5. Perceived Complexity of Access to Secondary Care

The process of accessing services was perceived as unclear and confusing for both refugees and NGOs. One KI noted that in 2016 many NGOs were unaware of how to refer into the secondary healthcare system. This problem was not unique to refugees alone:

*The main problem might be that actual healthcare services are not accessible, maybe because the Greek system is complicated. It is difficult for Greek people to access it. People have to get a paper, then get in, then go to another office, then another place. Then sometimes have to go by phone and don’t know how to do it as they can’t speak Greek. It is a complicated system in terms of accessibility. It is not that they do not have access to it.—*KI 7

#### 3.2.6. Information Exchange among Stakeholders and with Patients

KIs highlighted the poor health information exchange between healthcare workers, with patients responsible for their own medical records and referral paperwork. NGOs were provided with little information as to how to refer into secondary care and often had to make their own links to develop pathways. KIs reported that community clinics for refugees were helpful as they could streamline referrals into the system. One KI noted:

*Knowing where to go [for healthcare] can be challenging [for refugees]. There are a lot of different hospitals here; military hospitals for example … This is not easily navigated or explained unless they have someone to help them. This is why community clinics are good because at least it is a first step and people have set up a referral pathway for them.—*KII 11

#### 3.2.7. Legal Barriers

To access the Greek national health system, refugees needed to obtain an AMKA number (social security number). Whilst many KIs mentioned the lack of this as a large barrier to healthcare access, opinions differed regarding the ease of obtaining this number. KI 12 described the process as complicated whereas KI 14 described it as, in most cases, smooth. Theoretically, everyone (including refugees) is entitled to this number, but “in some cases, you still have some bureaucratic flaws.” Lack of legal entitlement negatively impacted refugees as two participants recounted experiences of pregnant women in pain being refused care by healthcare workers in a hospital. 

#### 3.2.8. Distance to Health Facilities

KIs reported that distance to health facilities, particularly secondary or emergency care, impacted healthcare access and patient care. On the islands, most refugee camps were located at a distance away from the main towns, whereas hospitals were centrally placed. Refugees might have to travel 1–2 h to reach a large hospital, which was difficult as transport may not be available and language affected the ease of public transport use. As a result, refugees could be forced to rely on more expensive forms of transport including taxis. For NGOs, travelling 5 h to the camp and back meant less time was spent tending to refugee needs. Location also has other effects:

*In most cases, refugee camps are hidden and away from big cities and towns. They are in hiding. The implications of this is that they cannot be integrated into Greek society.*—KI 10

#### 3.2.9. Cost of Care

Expenses associated with healthcare were a huge burden for refugees and healthcare providers. Healthcare providers noted that costs of running projects in Greece was high compared to other settings and that employee costs added significantly to their running costs:

*Working in a European country is hugely expensive compared to working in other countries where you are normally based for emergencies. Just being able to get registered is very costly and time consuming, it is quite difficult…The cost of transport is expensive. We have to rent cars like a tourist would.*—KI 14

As the crisis became more protracted, raising funds to cover running costs became increasingly difficult:

*Compassion fatigue was certainly an issue. Initially it was very popular to be aware of the refugee crisis in the Middle East and in Europe and to interact with that, it was in the news and people were donating and compassionate towards the cause, which dies off very quickly when the next emergency happens or when people don’t want to be sad anymore …*—KI 3

The cost of medication in Greece was also noted to be a significant issue for refugees:

*Some electronic prescriptions have a contribution fee which is not much money but for some of the patients or refugees, is a significant amount*—KI 13

### 3.3. Evolution of Healthcare Response

During 2015, some NGOs received EU ECHO funding to support the provision of refugee healthcare. However, a long-term strategy to integrate refugee healthcare provision into the local health system influenced a decision to direct funds to the Greek government. Some KIs favoured this integration to avoid running a parallel health system and to ensure sustainability long term:

*…Has become much more organised, much more streamlined in its progression, more urbanised. Services now have become more centralised, based on polyclinics in towns that people can access from wherever they are living rather than [clinics] being in-camp or in refugee communities. [Services have] also been a lot more formalised and regulated—[this is a] positive thing. [There are a] lot less providers, those providers that are here have become more scrutinised, and have more targets to meet—more accountability. [There are] still a lot of issues that need to be addressed fairly urgently*—KI 3

However, some KI’s reported that the Greek government was currently unprepared for this strategy and further issues arose:

*After summer 2017, when funding started going to the government, a lot of NGOs started to leave. Then an urgent need on the islands occurred as the government could not handle the situation*— KI 12

*…The situation now is worse than it was last year. The gaps are getting wider, the population that has been there for longer is getting sicker, there is a bigger population and less actors, the mathematics of it is not looking good. We have actually scaled up our operation when they wanted to be scaling down, but they had to do the opposite*—KI 15

#### 3.3.1. Weak Co-Ordination Mechanisms

Many KIs commented that improved coordination was required at all levels including between NGOs themselves, between NGOs and the government, and with international organisations. Early in 2015, the lack of coordination was described by one KI as being like the *“wild west”*. This resulted from the number of NGOs and independent volunteers working on the islands with a lack of regulation and poor communication.

*During the peak of the crisis when the awareness was at its highest, in 2015/2016, there was a lot of money flowing, a lot of services being provided, not always in a co-ordinated manner… there was a lot of service provision, perhaps even too much, there were a lot of actors offering similar services…You had 2 or 3 health actors offering services around the clock at a campsite compared to normal living situations where you don’t have 24/7 primary health care*—KII 9

This was also the case in other parts of Greece. Poor co-ordination impacted on continuity of care and planning:

*We wouldn’t be told that camps were closing until pretty much the day of. This make it hard to plan as an organisation, but it is also hard to reassure people if you do not know what is happening yourself. A lot of volunteers that were with them built up a good relationship, particularly those who spoke Arabic with the refugees, there was a good trust level. But then that suddenly disappears, or they promise to be there the next day but cannot go because they have been moved to another camp. That trust then immediately drops*—KI 14

#### 3.3.2. Healthcare Gaps

Co-ordination issues resulted in duplication of services and some gaps in required healthcare. Most KIs reported mental health services as a major gap, which resulted from a combination of the poor living conditions and legal status:

*They often experienced many years of conflict within Syria and then also have the traumatic journey across sea. But the main traumas, in my experience, came from the borders being closed. They had in their mind that they were moving somewhere and going on so had a hope*—KI 2

Mental health professionals are in short supply in Greece, adding to the disease burden:

*In Athens, a city of 5 million, there are only 2 functioning psychiatric hospitals*—KI 13

Dental services were also reported to be in short supply with a large need among the refugees. Other gaps included maternal and child health and vaccination programs. KIs stated that maternal care was provided when midwives or obstetricians were available or in some cases was subsidized by humanitarian organisations for private care. 

#### 3.3.3. Transition from an Emergency to a Long-Term Response

The early focus on acute conditions including infectious diseases and traumatic injuries reflected the nature of a population on the move. As the crisis became more prolonged, non-communicable diseases became more of a concern both as new and established diagnoses; this included the increasing burden of mental health. The health profile of the population changed with time:

*After the EU–Turkey deal, the population became “stranded” in Greece and on the islands… Some patients have been there for 2 years now, so their needs have dramatically changed*—KII 15

## 4. Discussion

This study highlights some of the challenges which refugees face in accessing healthcare in Greece; some challenges are similar to those seen in other contexts whereas others are unique in Greece. The increase in arrivals of refugees to Greece coincided with the ongoing economic crisis in Greece, which has adversely affected it health system. This has not only affected the health system’s ability to meet the needs of refugees but also its local population. Challenges are heightened by differences in socio-cultural and language between the predominantly Syria, Afghani and Iraqi refugees and the local Greek population. In addition, weak coordination among stakeholders and poor information sharing among stakeholders and with patients affected the provision of healthcare for refugees [19]. 

The main emerging themes were (i) influence of socio-cultural factors including language and expectations of healthcare by the refugees, (ii) factors relating to access and integration into the Greek health system and (iii) evolution of the humanitarian response including changes to funding arrangements and the transition from an emergency to a long-term response. 

### 4.1. Socio-Cultural Factors

Socio-cultural factors affected both access to and satisfaction with healthcare services, for both providers and patients. Our finding that translation services in Greece in the period studied were insufficient to support refugees mirrors evidence from other settings [28]. These shortfalls could contribute to poor understanding of refugees’ concerns, their ability to understand the outcomes of the consultations, frustrations of being misunderstood and, consequentially, poor satisfaction with services [18,20,29]. Though humanitarian organisations like *Medecins sans Frontieres* (MSF) as well as the Greek Ministry of Health tried to employ translators (as well as cultural mediators [30]), they were still not available in sufficient numbers to meet the needs and for healthcare consultations, particularly in secondary care. In Greece, the range of spoken languages presented additional challenges. In contrast, Syrian refugees in Lebanon and Jordan—where Arabic is also spoken—may be less likely to face this particular challenge [31,32] compared with Syrian refugees in Turkey [33]. Frustrations relating to language barriers are not only felt by the refugees but also by healthcare providers [34]. 

Addressing these linguistic and cultural barriers is important but difficult. Interventions could include the provision of sufficient interpreters, setting up telephone interpretation services or other technologies, e.g., NaTakallam [35], which could ease this challenge. Alternatively, policymakers might consider lessons from the Turkish model in which Syrian healthcare workers have been permitted to re-train as primary healthcare physicians and provide healthcare only to fellow refugees [36]. This could reduce strains on the Greek health system, provide employment opportunities for refugees who are already healthcare workers and provide socio-culturally relevant healthcare. However, without sufficient planning, the risk of developing a parallel health system for refugees as has occurred in other settings, as well as challenges surrounding the registration of healthcare workers and the provision of resources to train and appropriately accredit refugee health workers for this context is costly and labour intensive. 

The predominantly Muslim refugee population discussed in this study presented challenges to the Greek healthcare workers who were unaware of some of the religious and cultural norms affecting health, and gender-related preferences for health service delivery. These barriers to effective care delivery were significant. Improving the availability of cultural mediators (who could also act as translators where possible) could help address this, as piloted in a project led by *Medecins sans Frontieres* (MSF) during the study period [18]. Enhancing awareness of multiculturalism through on-line [34] or in person workshops could support improved relationships between healthcare providers and refugees [37]. Again, Syrian or Iraqi refugees may not face the same socio-cultural factors in Lebanon or Jordan where the local population and healthcare providers share a similar culture and religion [38].

Linguistic and socio-cultural barriers also influenced ability to access the health system. This has been documented in other settings [19,39], but KIs perceived Greece’s health system to be particularly challenging to navigate. Logistical difficulties could add to this as refugee camps were mostly situated on the outskirts of towns and at some distance to the secondary healthcare facilities.

### 4.2. Responsiveness of Greece’s Health System

A program of primary healthcare reform in Greece was introduced in 2015 with support from WHO EURO with the aim of supporting universal health coverage [40]. Despite this, primary healthcare for refugees in Greece in the public health system remains under-developed, both for local populations and for refugees. The Greek government worked with humanitarian organisations to provide primary healthcare for refugees in the refugee camps [18] with referrals into the Greek public health system for specialist services for refugees with AMKA cards [41]. However, these AMKA cards could in practice be difficult for refugees to obtain.

The impact of Greece’s economic crisis on its health system has been described [42,43] and has also affected care for Greece citizens who have some of the highest out of pocket expenditures for healthcare in Europe [18,23,44]. For most refugees, this private co-payment is not an available option. They could also be subject to further costs such as prescription fees and transport due to the distance of camps from health facilities even if the healthcare consultations themselves are free. 

An integrated approach to primary healthcare for refugees could strengthen primary healthcare for both refugees and citizens and also support Greece’s compliance with the Sustainable Development Goals [45]. Similarly, public health interventions relevant to both refugees and citizens in Greece would be strengthened [46]. Movements in this direction would, however, need to be supported by trust-building measures and education to ensure acceptability among refugee populations who may have been accustomed to accessing secondary care services even for routine health needs.

### 4.3. Evolution of the Health Response

KIs noted that the health needs of refugees and the health and humanitarian response for refugees in Greece changed over time, a common feature as acute humanitarian crises become chronic. In 2015, the response was predominantly an emergency-based response with refugees moving quickly through Greece to reach their intended European destinations, including Germany and Scandinavia. The closure of the FYROM border in March 2016 substantially reduced onwards progress. 

The necessary health system evolution was limited not only by uncoordinated and duplicated systems but also by competition between humanitarian actors and funding insecurity. Early in the response, international NGOs were supported by emergency funding from the European Commission, particularly for primary health care and psychosocial support [47]. In July 2017, as part of a strategy of local integration, funding was redirected to the Greek government. KIs reported that although this was a reasonable and sustainable approach, systems were not yet established to accommodate the large refugee population. The loss of financial aid meant many international NGOs were unable to cover the high costs of working in Greece and gradually ceased activity as a result. This reduced health services available, and volunteers and doctors in the health system [including small humanitarian organisations] found themselves further stretched in environments where they are already prone to burnout [48,49]. 

Despite significant financial investment, the Governmental response to healthcare for refugees in Greece has been heavily criticized [50]. Triaging the highest demands for healthcare services, e.g., maternal, mental healthcare, vaccination, public health messaging and then supporting these with sufficient translators (or relevant innovations) could support Greece to provide the necessary care for refugees and for its citizens. 

### 4.4. Weak Coordination 

Weak coordination amongst humanitarian organisations working on the response was highlighted by a number of KIs as a key factor in the response; it has also been highlighted in prior literature on humanitarian responses. [18,19,20]. These included poor communication among stakeholders, which could lead to duplication of services for some health needs but insufficient services or poor geographical spread of other services. In Greece, challenges related to coordination were multifactorial; they resulted partly from the rapidly changing situation and evolution of the humanitarian response, the presence of new or unregulated humanitarian organisations who were set up in response to the arrival of refugees in Greece [51], the spread of refugees across the islands and mainland and lack of strategic clarity from the Greek government or main actors[18] [20]. In addition, the government institutions in Greece, a high-income country, may not have as much experience as low- and middle-income countries (LMICs) at engaging with external partners for domestic humanitarian crises. 

The rapid and significant increase of refugee arrivals during 2015 placed significant strains on Greece’s health system both on the islands (where most refugees first arrived) and the mainland [20]. The volunteer-driven nature of the response initially was likely due to the slow local and international mobilisation and the late arrival of established humanitarian actors [18] to meet the needs of these new arrivals. Poor coordination was noted to result in duplication in some geographical areas or health needs and gaps in others [52]. This was particularly the case for more challenging interventions, e.g., non-communicable diseases [53] or mental healthcare [54]. Interventions to improve coordination could include incentivising cooperation, e.g., through pooled funds or access to beneficiaries [55], ensuring clear leadership structures and accountability for humanitarian actors as well as reducing competition among key actors. This leadership and coordination could be led by the EU, given the ongoing socio-economic situation in Greece.

### 4.5. Strengths and Limitations of This Study

This study is one of few which draws on interviews with KIs who have worked on the health response for refugees in Greece and is also one of the first to directly address the question of a humanitarian response in a high-income setting. It corroborates some findings already documented in the literature, but it also introduces new themes into the discussion of healthcare access for refugees in Greece and elsewhere. The KIs interviewed are drawn from varied backgrounds and include representatives of both humanitarian and international organisations, therefore providing different perspectives. Analysis of transcripts was done using a structured approach, building upon themes identified from a preceding analysis of the literature in order to reduce subjectivity.

Limitations of this study include the relatively small sample size and the short timeline during which the KII’s were performed. Purposive sampling heightens the risk of selection bias in small studies and in this case, there were more interviews with humanitarian organisation workers than with those from other actors. In particular, two key constituencies are absent from the list of interviewees: [1] refugees themselves and [2] Greek health officials. These are important limitations because refugees’ experiences as service users would provide important insights on system responsiveness as well as linguistic and other barriers to access and because officials’ views would have given a better sense of governance and financing constraints under which aspects of the response have operated. These groups were not consulted because of the stringent ethics approval requirements involved and time constraints. As with all qualitative (interview based) research, some of the information may be subject to recall or reporting bias. This risk was mitigated by cross checking among the KIs and also available literature, as well as through the use of anchoring terms in the interview topic guide to clarify which period of time respondent answers referred to. Most of the KIIs were performed in Athens, and this may not be representative of the situation across the rest of Greece, although the perspectives and experience of KIs represented a wider geographical region across Greece. Finally, given the scope of this study, thematic analysis was performed by only one reviewer.

## 5. Conclusions

Among the many barriers to healthcare access for refugees in Greece, the most noted were socio-cultural and language diversity as well as weak coordination among stakeholders. In addition, the inconsistent integration of refugees into the Greek health system is a missed opportunity to improve the health system for both refugees and Greek citizens. Policies and interventions that address these barriers could support healthcare access for refugees in Greece.

## Figures and Tables

**Table 1:** This table summarises the details of the key informants.

Location	*N* = 16	%
London, England	2	13
Athens, Greece	7	44
Skype/Phone	7	44
Gender		
Male	7	44
Female	9	56
Age		
<30	4	25
30–39	8	50
40–49	3	19
>50	1	6
Number of years working on Greece response		
<1 year	2	13
1–2 years	6	38
>2 year	8	50
Organisation		
NGOs * (local and international)	11	69
International organisations *	5	31

* Participating organisations have been de-identified.

## Abbreviations

EU	European Union
UNHCR	United Nations High Commissioner for Refugees
WHO	World Health Organisation
FYROM	Former Yugoslav Republic of Macedonia
NGO	Non-Governmental Organisation
MSF	Médecins Sans Frontières
MdM	Médecins du Monde
HS	Health System
PHC	Primary Health Care
KII	Key Informant Interview
KI	Key Informant
MoH	Ministry of Health
AMKA	Αριθμός Μητρώου Κοινωνικής Ασφάλισης (Greek social security number)
ECHO	European Civil Protection and Humanitarian Aid Operations
NCD	Non-Communicable Diseases

## Appendix A

### Appendix A.1. Questionnaire

BASIC INFORMATION

(1) What is your role in your organisation?
(2) Can you tell me what training you have and about prior experience in similar contexts?
(3) How long have you worked on the Greece response?

ABOUT ORGANISATION

(4) Name of organisation
(5) What is the role of the organisation?
(6) How long has it been operational in Greece?
(7) How big is it? E.g.,
(8) How many people are employees in Greece?
(9) How many refugees do you serve?
(10) How many consultations?
(11) What sectors, e.g., MHPSS/MCH/WASH/Shelter, etc. does it work in?
(12) Where in Greece does it operate?
(13) How has its work changed over time, e.g., scope and location?

PROVISION OF HEALTHCARE TO REFUGEES

(14) Who provides primary health care and how has this changed?
(15) Who provides secondary health care and how has this changed?
(16) Who provides speciality care: maternal and child health/psychosocial/pre-natal/dental?
(17) What are the main gaps in service provision in your opinion?
(18) What are the main health issues observed among refugees in Greece? What is not adequately provided for?

HEALTHCARE ACCESS

(19) Are refugees satisfied with what is provided in primary care?
(20) Are refugees satisfied with what is provided in secondary care?
(21) What are the main barriers to accessing care for refugees in Greece, e.g., Primary/secondary/emergency/maternity?
  (a) Physical accessibility
  (b) Language
  (c) Cultural factors determining care-seeking behaviour
  (d) Cost of care
(22) What are the main challenges in providing health care to refugees?
(23) What initiatives are adopted to overcome certain challenges?

CHANGES OVER TIME

(24) How has health service provision changed during your time working in Greece?
(25) What improvements do you think could be made to the provision of healthcare in Greece?
(26) How have the roles of government/NGOs/International agencies changed over the period 2015–2017 and what has influenced these changes?

## Appendix B

Using the framework method for thematic analysis (further details).

(1)  TRANSCRIPTION

Verbal consent was obtained prior to each interview for audio recording. On completion of the interviews, interviews were transcribed by the interviewer. During transcriptions, audio-recordings would be repeated to reduce errors.

(2)  FAMILIARISATION

After all interviews had been transcribed, the transcripts were re-read multiple times, so the content was familiar to the reviewer. During the familiarisation process, initial themes were elicited and noted down. These themes would include aspects that were mentioned by the majority of key-informants, e.g., the significance of a lack of translators in Greece. Additionally, when key informant perspectives differed on a particular topic, this was also noted. Due to the vast amount of text generated by the interviewees, the familiarisation process aided the reviewer to navigate the transcripts further on in the process.

(3)  CODING

Themes identified during familiarisation of the transcripts were compared with initial themes identified in the literature search. Mostly, the themes identified from the literature review were mentioned by interviewees. Relevant sections of text relevant to each theme were underlined in the transcript. These sections ranged from a few words to whole paragraphs of text which adequately described or emphasised the nature of a particular theme. For example, whilst some paragraphs explaining the impact of poor co-ordination were outlined, so were descriptive passages of text, such as one key informant describing the situation as being like the “wild west”. In addition, throughout the coding process, more themes were identified. Many quotes seemed to belong under specific headings but also relevant to over-arching themes, for example, many interviewees mentioned the impact of a refugee’s culture on their expectation of healthcare; codes underlying each theme were elicited and organised.

(4)  DEVELOPING A WORKING ANALYTICAL FRAMEWORK

After identifying initial themes and codes, they were reviewed again. After initial coding, there was a very comprehensive list, not allowing for meaningful discussion of causes and their place in the wider literature. Therefore, a final list of themes and codes was deducted in order to highlight the most significant findings and focus on the most important themes and codes mentioned in the key informant interviews. It was noted that many over-arching themes covered varying aspects. Therefore, codes were further elicited, underlying relevant themes—allowing for further discussion on their individual roles in this health response. As a result, there were three emerging themes each with a number of relevant, underlying codes. The final list was discussed amongst the authors regarding their suitability in capturing the main findings in the interviews pertaining the Greek refugee health response.

(5)  APPLYING THE ANALYTICAL FRAMEWORK

Once a final analytical framework had been formed, the transcripts were systematically reviewed in order to highlight passages relevant to each theme and code. A section of the highlighted transcript is shown below.

(6)  CHARTING DATA INTO THE FRAMEWORK MATRIX

On completion of coding all the transcripts as per the framework, a Word document was used to organise passages of text under each theme for ease of interpretation as displayed below. Due to the anonymization of the data, codes were assigned to a key-informant number. This produced a comprehensive list of “quotes” relevant to each theme. For discussion, it was important that the clearest excerpts were used or passages which explained certain deficits in the health response. As such, KII 15 described: “If you can do good primary healthcare, everything else becomes easier, you do not need really strong referral services to secondary care if you do good primary health care”. This passage was included as it discussed the reason why the lack of primary health care limited the health response in Greece. Many key informants mentioned that the lack of primary health care was an issue but did not delve into why.

(7)  INTERPRETING THE DATA

The desk-based literature review not only shaped the interview schedule but also helped identify initial themes. Most of the themes identified in the literature were corroborated by the key informants. What the literature review highlighted the most was the lack of literature regarding important aspects that influenced the health response to refugees in Greece. These findings were the most significant as were mechanisms as to why. Further literature was reviewed to identify whether these issues were present in other humanitarian crises outside Greece.
